# The Immunogenicity of DENV1–4 ED3s Strongly Differ despite Their Almost Identical Three-Dimensional Structures and High Sequence Similarities

**DOI:** 10.3390/ijms24032393

**Published:** 2023-01-25

**Authors:** Md. Din Islam, Tahmina Sharmin, Imrul Hasan Tipo, Antara Saha, Sanjida Yesmin, Moushumi Ghosh Roy, Subbaian Brindha, Yutaka Kuroda, M. Monirul Islam

**Affiliations:** 1 Department of Biotechnology and Life Science, Faculty of Engineering, Tokyo University of Agriculture and Technology, 2-24-16 Nakamachi, Koganei-shi, Tokyo 184-8588, Japan; 2 Department of Biochemistry and Molecular Biology, Faculty of Biological Sciences, University of Chittagong, Chittagong 4331, Bangladesh; 3 Department of Biotechnology, Lovely Professional University, Jalandhar 144001, India; 4 Institute of Global Innovation Research, Tokyo University of Agriculture and Technology, 3-8-1 Harumi-cho, Fuchu-shi, Tokyo 183-8538, Japan

**Keywords:** dengue envelop protein, immunogenicity, anti-ED3 IgG, effector T-cell memory, DENV serospecificity, DENV sero-cross-reactivity

## Abstract

The development of a dengue (DENV) vaccine remains challenging due to the heteroserotypic infection, which can result in a potentially deadly hemorrhagic fever or dengue shock syndrome, and only a tetravalent vaccine can overcome this issue. Here, we report the immunogenicity of DENV envelope protein domain 3 (ED3) from all four DENV serotypes (DENV1–4) in Swiss albino and BALB/c mice models. Firstly, we observed that despite having very similar sequences and structures, both the humoral and cellular immunogenicity of ED3s varied significantly, with strength ranging from DENV2 ED3 (2ED3)~3ED3 > 1ED3 > 4ED3, which was assessed through anti-ED3 IgG titers, and DENV1 ED3 (1ED3) > 2ED3~3ED3 > 4ED3 as determined by monitoring T-cell memory (CD44+CD62L+ T cells with IL-4 and IFN-γ expression). Secondly, anti-1ED3 sera cross-reacted with 2ED3 and 3ED3; anti-2ED3 and anti-3ED3 sera cross-reacted with each other, but anti-4ED3 was completely serotype-specific. The lack of reciprocity of anti-1ED3’s cross-reaction was unanticipated. Such disparity in the ED3 responses and cross-reaction might underlie the appearance of hemorrhagic fever and dengue shock syndrome. Hence, the development of an ED3-based tetravalent subunit vaccine would require understanding the aforementioned disparities.

## 1. Introduction

Dengue fever is the world’s most recurrent mosquito-borne viral disease, and almost 40% of the world’s population lives in dengue-endemic regions [1,2]. Over 390 million infections are reported yearly, with 25–33% requiring clinical treatment [3,4]. Moreover, dengue has become endemic in most tropical countries, with increased disease incidence and severity, especially in developing countries [5].

Dengue virus is classified into four serotypes (DENV1–4) [6,7,8]. Primary DENV infection by a serotype causes high fever (dengue fever; DF), but most patients recover and may gain a long-lasting immunity against the infecting DENV serotype [9,10]. However, secondary infection by a heteroserotypic DENV can cause severe dengue hemorrhagic fever (DHF) and dengue shock syndrome (DSS) [11,12]. DHF and DSS are caused by antibody-dependent enhancement (ADE), wherein the sub-neutralizing primary anti-DENV antibodies are weakly neutralizing against the heteroserotypic DENV and are incapable of virus clearance [13]. In dengue hyperendemic locations, all four DENV serotypes may co-circulate, generating a high prevalence of DHF and DSS [14,15]. Concurrent or multiple DENV infections by distinct serotypes complicate the understanding of the dengue disease etiology and, hence, limit the development of dengue vaccines [14]. 

The DENV genome is composed of a single open reading frame encoding five non-structural proteins and three structural proteins: the capsid protein, membrane protein, and envelope protein (E-protein) [16]. The E-protein is dimeric, where each monomer comprises three domains: ED1, ED2, and ED3 [17]. Domain 3 (ED3) has been reported to be involved in virus attachment to the host cells and includes the putative epitope residues on its surface [18,19]. Furthermore, monoclonal antibodies produced against ED3s could prevent viral attachment to the host cell, making it a prime candidate for developing anti-DENV therapeutics [19].

In this study, we examined the antibody responses against DENV1–4 ED3s in Swiss albino and BALB/c mouse models through immunization with ED3s. Here, we observed that despite having sequence homology and almost identical structures, both the humoral and the cellular immune response intensity were strongly serotype-dependent, and the anti-sera cross-reaction was also disparate. We discuss how such strong disparity in the ED3 responses and cross-reaction might influence the appearance of hemorrhagic fever and dengue shock syndrome.

## 2. Results

### 2.1. Serospecificity of Anti-DENV ED3 Sera 

**Figure 1:**
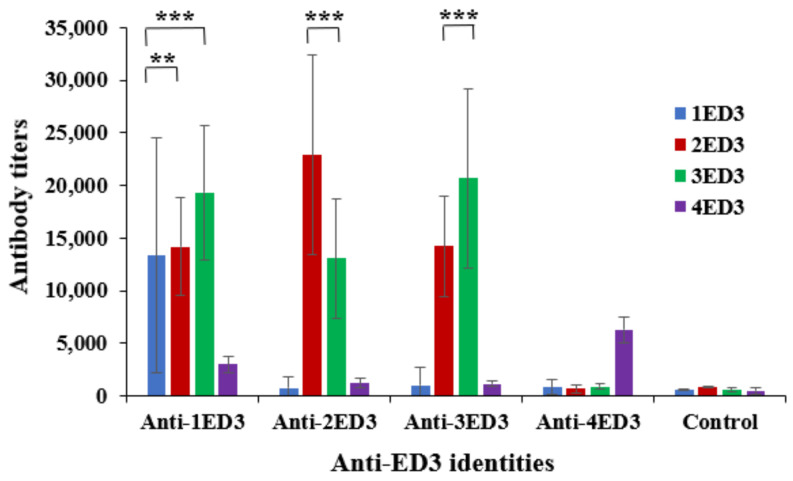
The serospecificity, sero-cross-reactivity, and immunogenicity of DENV1–4 ED3s in mouse models. Four different groups of mice were injected with four different ED3s in complete adjuvants in BALB/c mice. Anti-ED3 IgG titers on day 28 following 1st immunization are shown. First, all four ED3s were immunogenic and generated serotype-specific anti-ED3 IgG responses. Second, anti-1ED3 IgG was 1ED3-2ED3-3ED3 cross-reactive, and anti-2ED3 and anti-3ED3 were 2ED3-3ED3 cross-reacting. In contrast, anti-4ED3 was completely serotype-specific (4ED3-specific) with no-cross-recognition of other ED3s. Asterisks represent the comparisons using Dunnett’s multiple comparison test; ** *p* < 0.001 and *** *p* < 0.0001.

The serospecificity of anti-ED3 sera was tested by ELISA against the respective ED3 used as the coating antigen. The sero-specific anti-ED3 IgG titers of anti-ED3 sera clearly indicated that, despite being a small domain of the whole DENV E-protein, all four ED3s generated DENV ED3-specific IgG responses (Figure 1) [20]; anti-1ED3, -2ED3, -3ED3, and -4ED3 could recognize 1ED3, 2ED3, 3ED3, and 4ED3, respectively. However, serotype-specific antibody titers of anti-2ED3 sera and anti-3ED3 sera were similar and higher than those of anti-1ED3 sera, and antibody titers of anti-4ED3 sera were the lowest. These observations suggested that the immunogenicities of 2ED3 and 3ED3 were similar, and superior to those of 1ED3, and that 4ED3 was the least immunogenic ED3 (Figure 1 and Figure S1). 

### 2.2. Sero-Cross-Reactivity of Anti-DENV ED3 Sera 

The sero-cross-recognition of ED3s by other anti-ED3 sera was tested by ELISA using ED3s from a different serotype to the coating antigens. The anti-1ED3 sera showed sero-cross-recognition of both 2ED3 and 3ED3, but no cross-recognition of 4ED3 (Figure 1 and Figure S1). Interestingly, the antibody titers of anti-1ED3 against 2ED3 and 3ED3 were much higher than those against 1ED3 (Figure 1). The anti-2ED3 and -3ED3 sera were mostly serotype-specific with moderate cross-recognition of 3ED3 and 2ED3, respectively, but no cross-recognition of 1ED3 and 4ED3 (Figure 1 and Figure S1). In contrast, anti-4ED3 sera were solely 4ED3-specific with minimal or no cross-recognition of other ED3s (Figure 1 and Figure S1).

### 2.3. Long-Term Antibody Response against ED3s

**Figure 2:**
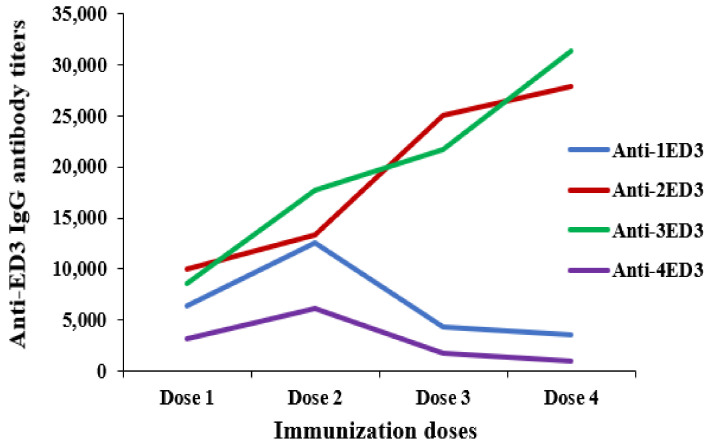
Long-term anti-ED3 IgG responses in BALB/c mouse model. Dose-specific anti-1ED3, anti-2ED3, anti-3ED3, and anti-4ED3 IgG responses (antibody titers) are shown. The sampling was done 28 days after each immunization with a 35-day interval between immunizations. The anti-ED3 IgG responses increased following the first two doses but decreased following doses 3 and 4 with 1ED3 and 4ED3. However, the dose-specific anti-2ED3 and anti-3ED3 IgG titers levels increased with repeated immunization doses.

The induction of long-term immunity against ED3s was monitored by measuring anti-ED3 IgG responses for 4 weeks after immunization (first dose) and following 3 additional intraperitoneal doses. The repeated injection of 2ED3 and 3ED3 followed a classical immune response pattern; the anti-2ED3 and anti-3ED3 IgG levels increased with the increasing number of doses (Figure 2). However, although anti-1ED3 and anti-4ED3 IgG levels increased with the first two doses, doses 3 and 4 did not increase IgG levels; the patterns of observed IgG titers decreased (Figure 2). Thus, not only did the four ED3s differ in immunogenicity, but they also differed in immunological memory. Specifically, the immune memory upon injection with 2ED3 and 3ED3 was sustained for two months. In contrast, it lasted only two weeks after injection with 1ED3 and 4ED3 (Figure 2).

### 2.4. CD Markers and Cytokines Expression

**Figure 3:**
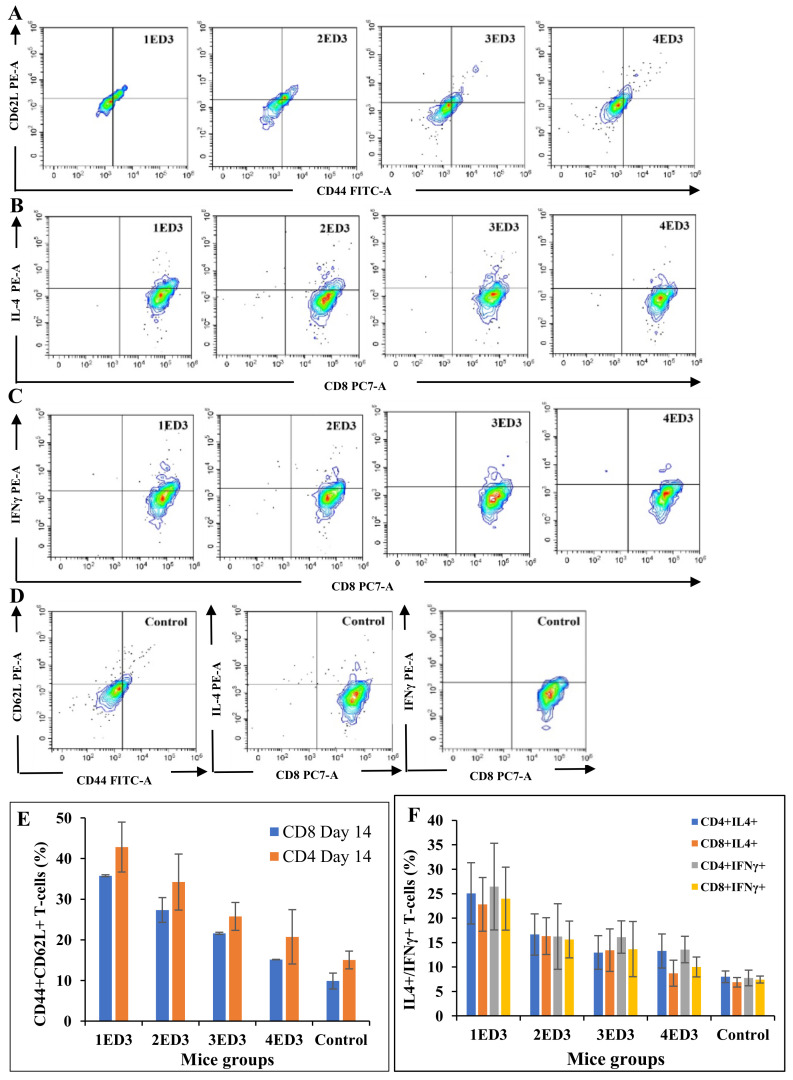
Effects of ED3 immunization on T-cell memory status in BALB/c mice. The CD44-CD62L co-expression at day 14 (**A**), IL-4 expression at day 7 (**B**), and IFN-γ expression at day 7 (**C**) by T_C_ cells are shown. Expression of CD44/CD62L, IL-4, and IFN-γ in unimmunized control mice (**D**). The relative percentages of CD44^+^CD62L^+^ T cells, and percentages of T cells expressing IL-4 and IFN-γ in ED3 immunized mice are shown in panels (**E**,**F**), respectively. The 1ED3 immunization resulted in the highest co-expression of CD44 and CD62L on T cells with the highest intracellular expression of IL-4 and IFN-γ by T cells, followed by 2ED3, 3ED3, and 4ED3 immunizations. These results suggested that 1ED3 was the most immunogenic while DENV4 ED3 was the least immunogenic ED3.

**Figure 4:**
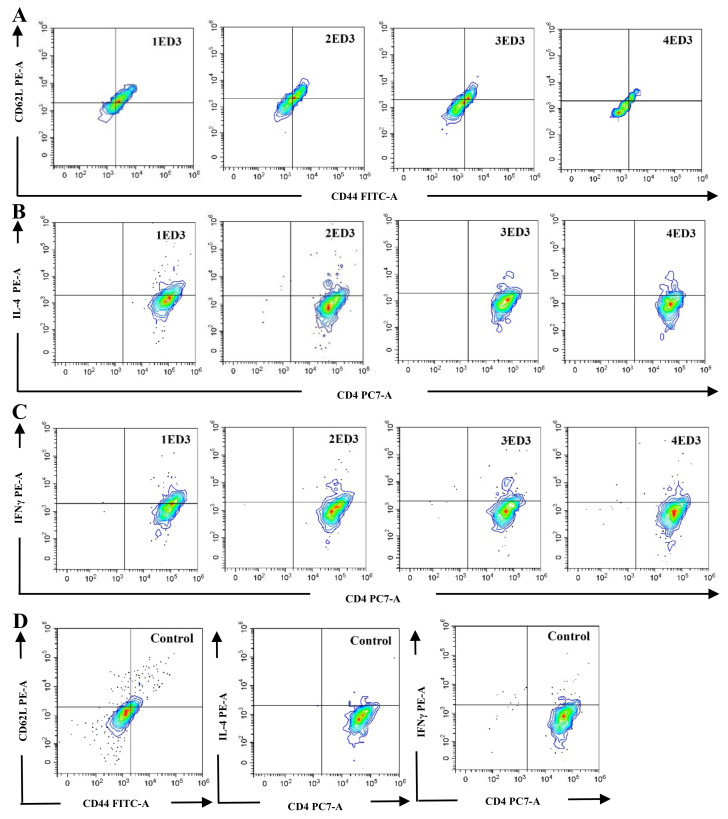
Effects of ED3 immunization on T-cell memory status in BALB/c mice. The CD44-CD62L co-expression at day 14 (**A**), IL-4 expression at day 7 (**B**), and IFN-γ expression at day 7 (**C**) by T_H_ cells are shown. Expression of CD44/CD62L, IL-4, and IFN-γ in unimmunized control mice (**D**).

To assess the effects of ED3 immunization on the expression of CD markers (CD3, CD4, CD8, CD44, and CD62L), and the intracellular expression of IL4 and IFN-γ, we monitored the differential expression in circulating T cells after the first dose of immunization. Immunization with 1ED3 generated the highest populations of CD44^+^ and CD44^+^CD62L^+^ T cells with the highest levels of IL-4 and IFN-γ in T-cells (both T_C_ and T_H_ cells), followed by 2ED3 and 3ED3 immunization (Figure 3 and Figure 4). In contrast, immunization with 4ED3 generated a minimal number of CD44^+^ and CD62L^+^ T_C_ and T_H_ cells with the lowest expression of IL-4 and INF-γ, at similar levels to the control mice (Figure 3 and Figure 4). The number of memory T cells and the levels of cytokines gradually decreased over several weeks (Supplementary Figures S2–S4). 

## 3. Discussion

**Figure 5:**
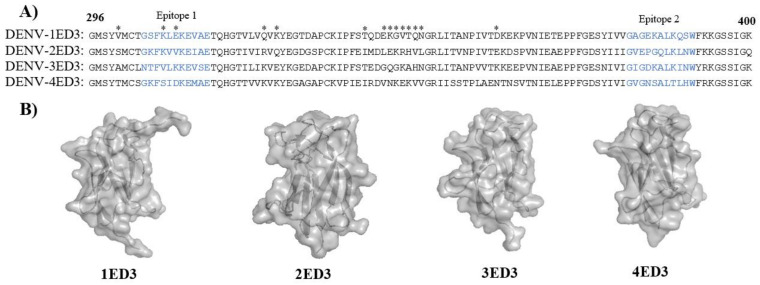
Sequences and structures of DENV ED3 variants. (**A**) The sequences 1ED3, 2ED3, 3ED3, and 4ED3 were retrieved from the UniProt IDs P17763, P14340, P27915.1, and P09866, respectively. The sequences were aligned using the online tool CLUSTAL W “www.ebi.ac.uk/Tools/msa/clustalo (accessed on 13 February 2022)” using the default settings [23]. The amino acid residues comprising epitope 1 and epitope 2 are marked in blue color. * The amino acid residues differ among the four different ED3s. (**B**) The surface structures models of 1ED3, 2ED3, 3ED3, and 4ED3 were generated from PBD IDs 3G7T.pdb [24], 4UTC.pdb [25], 3VTT.pdb [26], and 3WE1.pdb [27], respectively, using Pymol graphics “www.pymol.org (accessed on 13 February 2022)”. A few residues in the experimental sequence (Residues 296 and 381 in 1ED3 and 296 in 2ED3) described in the upper panel (**A**) differed from those in 3G7T.pdb (1ED3) and 4UTC.pdb (2ED3). The side chains were substituted in order to fit the sequence to those shown in (**A**) using COOT [28] and the Richardson rotamer library [29].

Despite its small size (11 kDa), ED3 of all four serotypes generated sero-specific IgG immune responses (Figure 2 and Figure S1). However, although the ED3s of DENV1-4 have very high sequence and structure similarities (Figure 5A,B and Figure S5; Supplementary Table S1), their immunogenicity, serospecificity, and sero-cross-specificity differed significantly [20,21,22]. 

The IgG responses against 2ED3 and 3ED3 were similar and higher than 1ED3 and 4ED3 (Figure 2). Furthermore, the anti-1ED3 sera were 1ED3, 2ED3, and 3ED3 cross-reactive, but anti-2ED3 and anti-3ED3 sera did not cross-recognize 1ED3. In contrast, the strict serospecificity and poor immunogenicity of DENV4 ED3, fully corroborating with a previous report where the immunogenicity of DENV4 was the least [22], could be considered as an intrinsic inherent property of 4ED3 [30]. In addition, immune memory generated following immunization with 2ED3 and 3ED3 sustained for two months. However, for 1ED3 and 4ED3, immune memory lasted only two weeks. Such heterogeneous immunogenicity of the four DENV ED3s might still be considered an obstacle to the limited success of available DENV vaccines [31]. Furthermore, the differential expression of CD markers on lymphocytes and the expression of intercellular cytokines by T cells following ED3 immunization also differed. More precisely, the highest number (%) of effector memory T cells were generated with the highest levels of IL-4 and IFN-γ expression by 1ED3. For this reason, 1ED3 was considered the most immunogenic in the induction of memory cells, followed by 2ED3, 3ED3, and 4ED3 (Figure 2 and Figure 3). The very low levels of CD44+CD62L+ T_C_ cells, along with low IL-4 and IFN-γ expression, confirmed their naive immunological status [32,33], while the high levels of CD44+CD62L+ T_C_ cells, with increased expression of IL-4 and IFN-γ, are an indication of long-term immunity through effector and central T-cell memory [34,35]. High expression levels of CD44 and CD62L on T cells may appear uncommon, but this has been observed in aerosol infection by Mycobacterium tuberculosis and choriomeningitis virus infection in mice [33,34,35,36,37,38,39,40]. Thus, the robust and persistent IgG responses, together with the high expression of CD44 and CD62L on T cells observed with all the ED3-variants except 4ED3, clearly indicated that the ED3s potentially allow efficient antigen uptake by antigen presenting cells [41], which is purported to enhance T-cell activation and antibody production [38].

## 4. Materials and Methods

### 4.1. Mutant Design Protein Expression and Purification

The ED3 sequences of DENV1, DENV2, DENV3, and DENV4 serotypes were retrieved from the UniProt database, and the nucleotide sequences, optimized for expression in *Escherichia coli*, were synthesized and cloned at the *NdeI* and *BamHI* sites of pET15b (Novagen) [26].

All ED3 variants were overexpressed in *E. coli* JM109(DE3)pLysS as inclusion bodies and refolded as described previously [42]. In short, after harvesting, the cells were lysed in lysis buffer (150 mM NaCl, 0.5% sodium deoxycholate, and 1% SDS in 50 mM Tris-HCl pH 8.5) and lysis wash buffer (lysis buffer supplemented with 1% *v/v* NP-40) through sonication. The cell lysates were air oxidized for 36 h at 30 °C in 6 M guanidine hydrochloride in 50 mM Tris-HCl, pH 8.7. The His_6_-tagged ED3s were purified by Ni-NTA (Wako, Tokyo, Japan) chromatography, followed by dialysis against 10 mM Tris-HCl, pH 8.0 at 4 °C. The N-terminal His_6_-tag was cleaved by thrombin proteolysis [43,44]. ED3s were purified by a second round of Ni-NTA chromatography followed by reversed-phase (RP) HPLC. The proteins were lyophilized and stocked as powder at −40 °C until use [21].

### 4.2. Analytical Reverse-Phase High-Performance Chromatography (HPLC)

The proteins were analyzed by reverse-phase (RP) high-performance liquid chromatography (HPLC; Shimadzu, Kyoto, Japan) using an Intrada 5WP-RP column (Imtakt, Kyoto, Japan), and absorbance at 220 nm was used to monitor the HPLC runs. Solution A (MilliQ-water + 0.1% trifluoroacetic acid (TFA)) and Solution B (Acetonitrile + 0.05% TFA) were used as a mobile phase with a flow rate of 1 mL/min and a column temperature of 30 °C. RP-HPLC analysis was performed using a 330 μL aliquot supplemented with acetic acid at a final concentration of 10% (*v/v*) and filtered with a 0.20 μm membrane filter to remove any aggregates. The reduced form of the protein was prepared by incubating the sample at pH 8.0 with 100 mM DTT for one hour at 37 °C, and the RP-HPLC analysis was performed as mentioned above [45].

### 4.3. Matrix-Assisted Laser Desorption/Ionization-Time of Flight Mass Spectroscopy (MALDI-TOF MS)

MALDI-TOF MS measurements were performed on an Autoflex speed TOF/TOF mass spectrometer (Bruker Daltonics, Billerica, MA, USA). The matrix solution was prepared by dissolving 10 mg of sinapic acid in 1 mL of Milli-Q water (M.Q.) with 0.1% Trifluoroacetic acid and 30% acetonitrile. The protein solution and the matrix solution were mixed to a 1:4 ratio, and 1 µL of the sample mixtures were spotted and air-dried on a MALDI-TOF MS plate [46]. 

### 4.4. Immunization Protocol

Artificial immunization studies against DENV1–4 ED3s were performed in five groups of Swiss albino and BALB/c (ICDDR, B, Dhaka, Bangladesh) mice aged 3–4 weeks at the start of the experiment. Immunization was conducted at 30 μg/dose/mouse in the presence and absence of Freund’s adjuvant [44,47]. In group 1 (20 Swiss albino, 5 mice for each group) and group 2 (20 BALB/c, 5 mice for each group), mice were injected with 1ED3, 2ED3, 3ED3, and 4ED3 in the presence of Freund’s adjuvant. Approximately 30 μg of ED3 was dissolved in 100 μL of phosphate-buffered saline (PBS, pH 7.4), and then 100 μL of Freund’s adjuvant was added to the protein solution and emulsified just prior to injecting into the mice (30 μg/200 μL/dose/mice). The first dose was given subcutaneously in Freund’s complete adjuvant, and the following doses were administered intraperitoneally at 35-day intervals in Freund’s incomplete adjuvant. In group 3 (12 mice) and 4 (12 mice), BALB/c mice were subjected to the same ED3 immunization study in the absence of any adjuvant (each dose comprising 30 μg of ED3 dissolved in 100 μL of PBS, pH 7.4). In group 5 (3 mice), the control group, BALB/c mice were injected only with PBS or PBS-adjuvant. Dose-specific anti-ED3 IgG responses were monitored against all four ED3s at weekly intervals for four weeks using an enzyme-linked immunosorbent assay (ELISA) [45]. Extracellular CD markers on circulating T-lymphocytes of the mice in group 3 (CD44 and CD62L) were monitored by flow cytometry on days 14 and 21 [8]. Mice in group 4 were monitored for intracellular cytokines (IL-4 and IFN-γ) on circulating T cells at weekly intervals for three consecutive weeks using flow cytometry [48]. 

### 4.5. Anti-ED3 IgG Titer by ELISA

The generation of anti-ED3 IgG antibody responses in mice was investigated by ELISA as previously described [43,49]. The 96-well microtiter plates (Dynatech Laboratories, EI Paso, TX, USA) were coated with 1 µg/mL of purified ED3s in PBS (100 µL/well) overnight at room temperature. Unbound proteins were washed away using PBS, and the plates were blocked with 1% BSA in PBS for 45 min at 37 °C. After washing with PBS, dose-specific mouse anti-sera were applied at 1:50 in 0.1% BSA in PBS, followed by a 3-fold serial dilution, and the plates were then incubated at 37 °C for 2 h. Unbound antibodies were removed by thoroughly washing three times with PBS-0.05% Tween-20 and once with PBS. Microtiter plates were blot-dried, and anti-mouse-IgG-HRP conjugates (Thermo Fisher Scientific, Waltham, MA, USA; 1:3000 dilution in 0.1% BSA-PBS-0.05% Tween-20) were added and incubated at 37 °C for 90 min. The unbound conjugates were removed by washing three times with PBS-0.05% Tween-20 and once with PBS. Coloring was performed by adding OPD (o-phenylenediamine) substrate at 0.4 μg/mL concentration supplemented with 4 mM H_2_O_2_ (100 µL/well). After 20 min of incubation at room temperature, the reaction was stopped by adding 50 µL of 1 N sulfuric acid, and the color intensity was measured at 450 nm (OD_450 nm_) using a microplate reader (Thermo Scientific Multiscan^®^ EX Primary EIA V2.3, Waltham, MA, USA). Antibody titers were calculated from the power fitting of OD_450 nm_ versus the reciprocal of the anti-sera dilution using a cut-off of OD_450 nm_ 0.1 above the background values. The ELISA data were analyzed using MS-Excel. 

### 4.6. Flow Cytometry Analysis of CD Markers

#### 4.6.1. Cell Surface CD Marker Analysis

To assess the effects of ED3 immunization on the expression of CD markers on T-lymphocytes, we monitored differential expression surface CD markers (CD3, CD4, CD8, CD44, and CD62L on circulating T cells) on days 14 and 21 following the first dose of immunization.

Whole blood collected through tail-bleed was mixed gently in FACS buffer (PBS supplemented with 2% FBS, 1 mM EDTA, and 0.1% sodium azide). The red blood cells (RBCs) were lysed with RBC lysis solution (0.15 M ammonium chloride, 10 mM potassium bicarbonate, 0.1 mM EDTA) for 5 min at room temperature and followed by washing twice with FACS buffer (1600 rpm, 4 °C, 5 min) and cells were resuspended in 100 µL pre-cooled FACS buffer. The cells were stained with anti-CD3-Pcy5, CD4-Pcy7, CD44-FITC, and CD62L-PE-conjugated antibodies (T_H_-cell lineage) and with anti-CD3-Pcy5, CD8-Pcy7, CD44-FITC, and CD62L-PE-conjugated antibodies (T_C_-cell lineage) (0.5 µg of antibodies/100 µL of cell suspension) for 30 min in the dark. Excess unbound conjugated antibodies were removed by washing the cells with FACS buffer. Finally, cells were resuspended in a 300 µL FACS buffer, and the data were collected using CytoFlex (Beckman Coulter, Brea, CA, USA). 

#### 4.6.2. Intracellular Cytokine Analysis

The effects of ED3 immunization on intracellular expression of IL4 and IFN-γ in circulating T cells (collected through tail-bleeding) were assessed on days 7, 14, and 21 following the first dose of immunization using the same procedure used for surface CD markers with 0.05% Tween-20 added, and the markers were labeled with fluorescence conjugated antibodies (CD3-Pcy5, CD4-Pcy7, and IL4-PE in one tube, and CD3-Pcy5, CD8-Pcy7, and INF-γ-PE in another tube). Flow cytometry data were collected from at least three mice, averaged, and presented with standard deviations.

### 4.7. Structure Modeling of Four Different ED3s

The structure models of 1ED3, 2ED3, 3ED3, and 4ED3 were generated from PBD IDs 3G7T.pdb [24], 4UTC.pdb [25], 3VTT.pdb [26], and 3WE1.pdb [27], respectively, using Pymol graphics “www.pymol.org (accessed on 13 February 2022)”. Briefly, the 3G7T.pdb (1ED3) and 4UTC.pdb (2ED3) were first modified in accordance with the sequences of 1ED3 and 2ED3 variants using COOT [28]. The Richardson rotamer library [29] was used for side-chain configuration for substituted residues. The sequences were aligned using the online tool CLUSTAL W “www.ebi.ac.uk/Tools/msa/clustalo (accessed on 13 February 2022)” with default settings [23]. All four ED3s were then superimposed, and models were generated using Pymol graphics. 

## 5. Conclusions

As confirmed by our study, the differential immunogenicity and sero-cross-behavior of anti-DENV IgG antibodies may be limiting factors for the success of tetravalent dengue vaccines. Here we also report, for the first time, that DENV1 is the most unusual among all DENV serotypes from the perspective of generating sero-specific and sero-cross-reactive anti-DENV antibody responses. Therefore, we suggest that future tetravalent dengue vaccine formulations must consider the immunogenicity of individual DENV serotypes and their sero-cross-talks with other anti-dengue antibodies in tetravalent compositions. We believe the present results represent a significant advance toward developing tetravalent dengue vaccines.

## Data Availability

Not applicable.

## Supplementary Materials

The following supporting information can be downloaded at: https://www.mdpi.com/article/10.3390/ijms24032393/s1.

## References

[1] Ashley E.A. (2011). Dengue fever. Trends Anaesth. Crit..

[2] Farrar J., Focks D., Gubler D., Barrera R., Guzman M.G., Simmons C., Kalayanarooj S., Lum L., McCall P.J., Lloyd L. (2007). Towards a global dengue research agenda. Trop. Med. Int. Health.

[3] Aguas R., Dorigatti I., Coudeville L., Luxemburger C., Ferguson N.M. (2019). Cross-serotype interactions and disease outcome prediction of dengue infections in Vietnam. Sci. Rep..

[4] Shepard D.S., Undurraga E.A., Halasa Y.A., Stanaway J.D. (2016). The global economic burden of dengue: A systematic analysis. Lancet.

[5] Murray N.E., Quam M.B., Wilder-Smith A. (2013). Epidemiology of dengue: Past, present and future prospects. J. Clin. Epidemiol..

[6] Rahman N., Miura S., Okawa M., Kibria M.G., Islam M.M., Kuroda Y. (2021). Solubility Controlling Peptide Tags of Opposite Charges Generate a Bivalent Immune Response Against Dengue ED3 Serotypes 3 and 4. Front. Immunol..

[7] Bhatt S., Gething P.W., Brady O.J., Messina J.P., Farlow A.W., Moyes C.L., Drake J.M., Brownstein J.S., Hoen A.G., Sankoh O. (2013). The global distribution and burden of dengue. Nature.

[8] Warrilow D., Northill J.A., Pyke A., Smith G.A. (2002). Single rapid TaqMan fluorogenic probe-based PCR assay that detects all four dengue serotypes. J. Med. Virol..

[9] Murphy B.R., Whitehead S.S. (2011). Immune response to dengue virus and prospects for a vaccine. Annu. Rev. Immunol..

[10] Imrie A., Meeks J., Gurary A., Sukhbaatar M., Truong T.T., Cropp C.B., Effler P. (2007). Antibody to dengue 1 detected more than 60 years after infection. Viral Immunol..

[11] Rothman A.L. (2004). Dengue: Defining protective versus pathologic immunity. J. Clin. Investig..

[12] Snow G.E., Haaland B., Ooi E.E., Gubler D.J. (2014). Research on dengue during World War II revisited. Am. J. Trop. Med..

[13] Wahala W.M., De Silva A.M. (2011). The human antibody response to dengue virus infection. Viruses.

[14] Recker M., Blyuss K.B., Simmons C.P., Hien T.T., Wills B., Farrar J., Gupta S. (2009). Immunological serotype interactions and their effect on the epidemiological pattern of dengue. Proc. Royal Soc. B.

[15] Halstead S.B., Nimmannitya S., Cohen S.N. (1970). Observations related to pathogenesis of dengue hemorrhagic fever. IV. Relation of disease severity to antibody response and virus recovered. Yale J. Biol. Med..

[16] Gebhard L.G., Filomatori C.V., Gamarnik A.V. (2011). Functional RNA elements in the dengue virus genome. Viruses.

[17] Gromowski G.D., Barrett A.D. (2007). Characterization of an antigenic site that contains a dominant, type-specific neutralization determinant on the envelope protein domain III (ED3) of dengue 2 virus. Virology.

[18] Slon-Campos J.L., Dejnirattisai W., Jagger B.W., López C.C., Wongwiwat W., Durnell L.A., Winkler E.S., Chen R.E., Reyes S.A., Rey F.A. (2019). A protective Zika virus E-dimer-based subunit vaccine engineered to abrogate antibody-dependent enhancement of dengue infection. Nat. Immunol..

[19] Rajamanonmani R., Nkenfou C., Clancy P., Yau Y.H., Shochat S.G., Sukupolvi P.S., Schul W., Diamond M.S., Vasudevan S.G., Lescar J. (2009). On a mouse monoclonal antibody that neutralizes all four dengue virus serotypes. J. Gen. Virol..

[20] Midgley C.M., Bajwa J.M., Vasanawathana S., Limpitikul W., Wills B., Flanagan A., Waiyaiya E., Tran H.B., Cowper A.E., Chotiyarnwon P. (2011). An in-depth analysis of original antigenic sin in dengue virus infection. J. Virol..

[21] Islam M.M., Miura S., Hasan M.N., Rahman N., Kuroda Y. (2020). Anti-Dengue ED3 long-term immune response with T-cell memory generated using Solubility Controlling Peptide tags. Front. Immunol..

[22] Lazo L. (2020). Dengue virus 4: The ‘black sheep’of the family. Expert Rev. Vaccines.

[23] Thompson J.D., Higgins D.G., Gibson T.J. (1994). CLUSTAL W: Improving the sensitivity of progressive multiple sequence alignment through sequence weighting, position-specific gap penalties and weight matrix choice. Nucleic Acids Res..

[24] Nayak V., Dessau M., Kucera K., Anthony K., Ledizet M., Modis Y. (2009). Crystal structure of dengue virus type 1 envelope protein in the post fusion conformation and its implications for membrane fusion. J. Virol..

[25] Kikuti C., Rouvinski A., Guardado C.P., Barba S.P., Barba S.G., Duquerroy S., Vaney M.C., Rey F.A. (2015). Crystal structure of dengue 2 virus envelope glycoprotein. Nature.

[26] Elahi M., Islam M.M., Noguchi K., Yohda M., Kuroda Y. (2013). High resolution crystal structure of dengue-3 envelope protein domain III suggests possible molecular mechanisms for serospecific antibody recognition. Proteins Struct. Funct. Genet..

[27] Elahi M., Islam M.M., Noguchi K., Yohda M., Toh H., Kuroda Y. (2014). Crystal structure of dengue 4 envelope protein domain III (ED3). Biochim. Biophys. Acta Proteins Proteom..

[28] Kulkarni M.R., Islam M.M., Numoto N., Elahi M., Mahib M.R., Ito N., Kuroda Y. (2015). Structural and biophysical analysis of sero-specific immune responses using epitope grafted Dengue ED3 mutants. Biochim. Biophys. Acta Proteins Proteom..

[29] Lovell S.C., Word J.M., Richardson J.S., Richardson D.C. (2000). The penultimate rotamer library. Proteins Struct. Funct. Genet..

[30] Ji G.H., Deng Y.Q., Yu X.J., Jiang T., Wang H.J., Shi X., Zhang D.P., Li X.F., Zhu S.Y., Zhao H. (2015). Characterization of a novel dengue serotype 4 virus-specific neutralizing epitope on the envelope protein domain III. PLoS ONE.

[31] Liu Y., Liu J., Cheng G. (2016). Vaccines and immunization strategies for dengue prevention. Emerg. Microbes Infect..

[32] Kaech S.M., Wherry E.J., Ahmed R. (2002). Effector and memory T-cell differentiation: Implications for vaccine development. Nat. Rev. Immunol..

[33] Bannard O., Kraman M., Fearon D. (2009). Pathways of memory CD8+ T-cell development. Eur. J. Immunol..

[34] Roberts A.D., Ely K.H., Woodland D.L. (2005). Differential contributions of central and effector memory T cells to recall responses. J. Exp. Med..

[35] Faassen V.H., Saldanha M., Gilbertson D., Dudani R., Krishnan L., Sad S. (2014). Reducing the stimulation of CD8+ T cells during infection with intracellular bacteria promotes differentiation primarily into a central (CD62LhighCD44high) subset. J. Immunol..

[36] Baaten B.J., Tinoco R., Chen A.T., Bradley L.M. (2012). Regulation of antigen-experienced T cells: Lessons from the quintessential memory marker CD44. Front. Immunol..

[37] Budd R.C., Cerottini J.C., Horvath C., Bron C., Pedrazzini T., Howe R.C., MacDonald H.R. (1987). Distinction of virgin and memory T lymphocytes. Stable acquisition of the Pgp-1 glycoprotein concomitant with antigenic stimulation. J. Immunol..

[38] Liang X., Li X., Duan J., Chen Y., Wang X., Pang L., Kong D., Song B., Li C., Yang J. (2018). Nanoparticles with CD44 targeting and ROS triggering properties as effective in vivo antigen delivery system. Mol. Pharm..

[39] Feng C.G., Bean A.G., Hooi H., Briscoe H., Britton W.J. (1999). Increase in gamma interferon-secreting CD8+, as well as CD4+, T cells in lungs following aerosol infection with Mycobacterium tuberculosis. Infect. Immun..

[40] Whitmire J.K., Asano M.S., Murali K.K., Suresh M., Ahmed R. (1998). Long-term CD4 Th1 and Th2 memory following acute lymphocytic choriomeningitis virus infection. J. Virol..

[41] Ahmadi M., Bryson C.J., Cloake E.A., Welch K., Filipe V., Romeijn S., Hawe A., Jiskoot W., Baker M.P., Fogg M.H. (2015). Small amounts of sub-visible aggregates enhance the immunogenic potential of monoclonal antibody therapeutics. Pharm. Res..

[42] Kato A., Maki K., Ebina T., Kuwajima K., Soda K., Kuroda Y. (2007). Mutational analysis of protein solubility enhancement using short peptide tags. Biopolymers.

[43] Kibria M.G., Akazawa O.Y., Rahman N., Hagihara Y., Kuroda Y. (2020). The immunogenicity of an anti-EGFR single domain antibody (VHH) is enhanced by misfolded amorphous aggregation but not by heat-induced aggregation. Eur. J. Pharm..

[44] Frei J.C., Wirchnianski A.S., Govero J., Vergnolle O., Dowd K.A., Pierson T.C., Kielian M., Girvin M.E., Diamond M.S., Lai J.R. (2018). Engineered dengue virus domain III proteins elicit cross-neutralizing antibody responses in mice. J. Virol..

[45] Brindha S., Kuroda Y. (2022). A Multi-Disulfide Receptor-Binding Domain (RBD) of the SARS-CoV-2 Spike Protein Expressed in E. coli Using a SEP-Tag Produces Antisera Interacting with the Mammalian Cell Expressed Spike (S1) Protein. Int. J. Mol. Sci..

[46] Saotome T., Yamazaki T., Kuroda Y. (2019). Misfolding of a single disulfide bonded globular protein into a low-solubility species conformationally and biophysically distinct from the native one. Biomolecules.

[47] Babu J.P., Pattnaik P., Gupta N., Shrivastava A., Khan M., Rao P.L. (2008). Immunogenicity of a recombinant envelope domain III protein of dengue virus type-4 with various adjuvants in mice. Vaccine.

[48] Kibria M.G., Akazawa-Ogawa Y., Hagihara Y., Kuroda Y. (2021). Immune response with long-term memory triggered by amorphous aggregates of misfolded anti-EGFR VHH-7D12 is directed against the native VHH-7D12 as well as the framework of the analogous VHH-9G8. Eur. J. Pharm..

[49] Rahman N., Islam M.M., Kibria M.G., Unzai S., Kuroda Y. (2020). A systematic mutational analysis identifies a 5-residue proline tag that enhances the in vivo immunogenicity of a non-immunogenic model protein. FEBS Open Bio.

