# Supplementary material for: The Immunogenicity of DENV1–4 ED3s Strongly Differ despite Their Almost Identical Three-Dimensional Structures and High Sequence Similarities
Source: Int J Mol Sci. 2023 Jan 25;24(3):2393. doi: 10.3390/ijms24032393 (PMC9916489; doi:10.3390/ijms24032393)
Supplement: Supplementary file 1 [file ijms-24-02393-s001.zip › ijms-2148147-supplementary.pdf]

Supplementary Data:

Table S1. Sequence and structural similarities among DEN ED3s.

	DENV1 ED3	DENV2 ED3	DENV3 ED3	DENV4 ED3
DENV1 ED3	-	0.78 Å[a]	0.89 Å	0.98 Å
DENV2 ED3	62.9%	-	0.69 Å	0.68 Å
DENV3 ED3	70.5%	59.1%	-	1.04 Å
DENV4 ED3	55.2%	60.0%	51.4%	-

[a] The numbers in the right top half and bottom left half correspond to the C-RMSD and percentage identity of two ED3 serotypes.

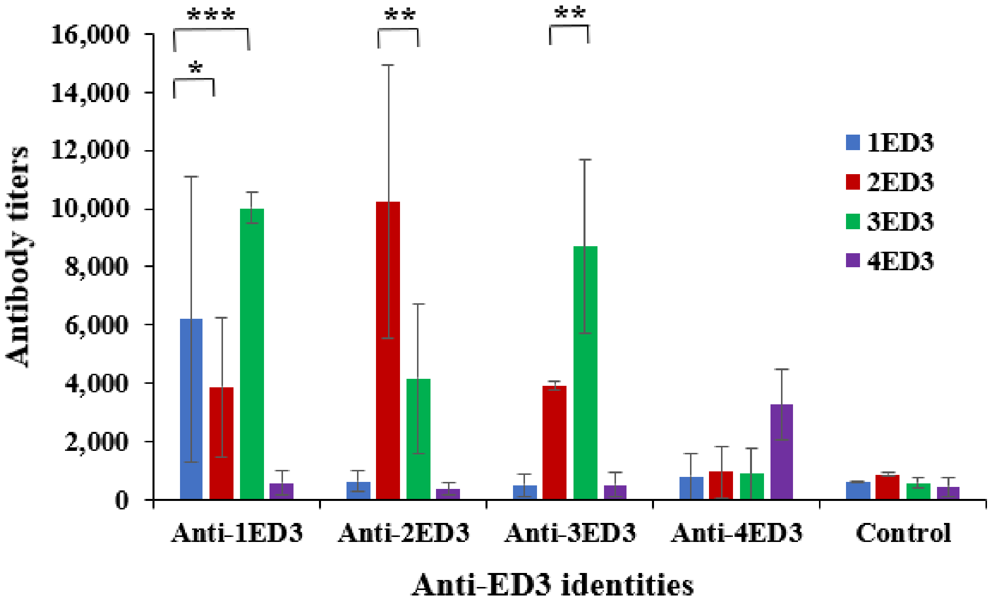

Figure S1. Anti-ED3 IgG antibody responses in Swiss Albino mice. This mice group was injected with four different ED3s as in Balb/c mice mentioned in results section. Here, the anti-ED3 IgG antibody responses on day 28 are shown. The results were similar as observed in the Balb/c mouse group. Asterisks represent the comparisons using Dunnett's multiple comparison test; **p<0.001, **p<0.001 and ***p<0.0001.

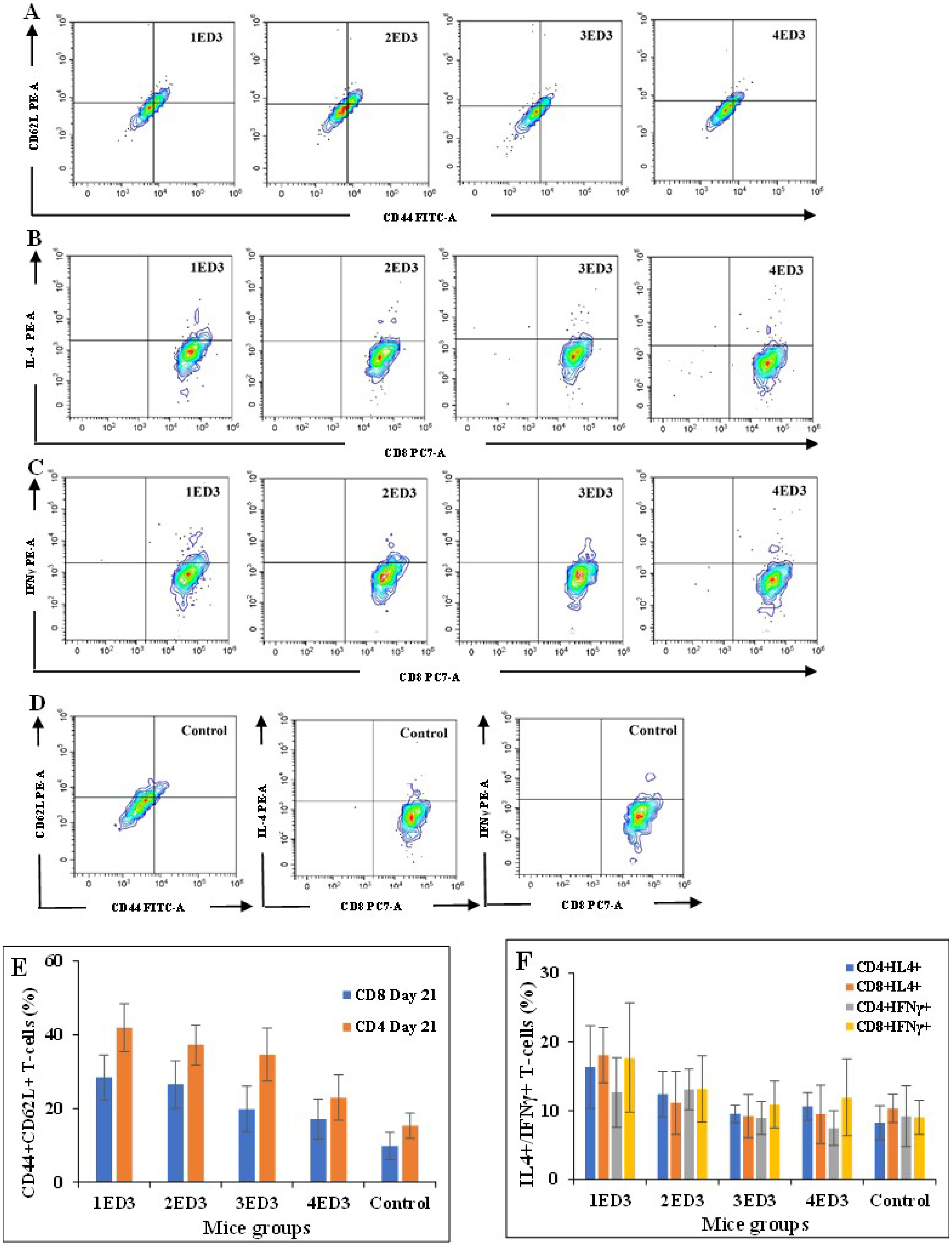

Figure S2. Effects of ED3 immunization on T-cell memory status in Balb/c mice. The CD44-CD62L co-expression at day 21 (A), IL-4 expression at day 14 (B) and IFN-γ expression at day 14

(C) by T_C-cells are shown. Expression of CD44/CD62L, IL-4 and IFN-γ in unimmunized control mice (D). The relative percentages of CD44+CD62L+T-cells, and percentages of T-cells expressing IL-4 and IFN-γ in ED3 immunized mice are shown in panels (E) and (F), respectively.

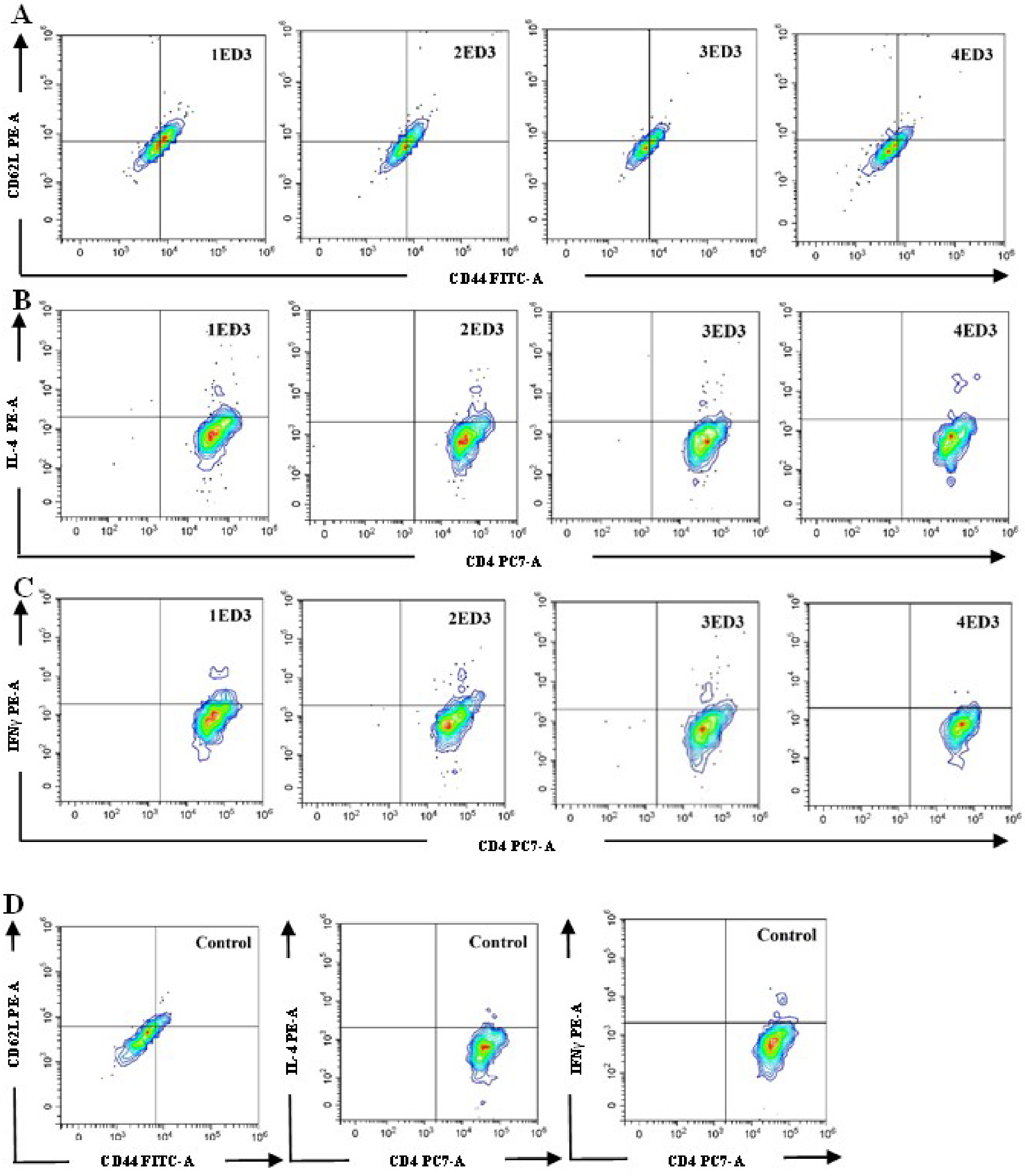

Figure S3. Effects of ED3 immunization on T-cell memory status in Balb/c mice. The CD44-CD62L co-expression at day 21 (A), IL-4 expression at day 14 (B) and IFN-γ expression at day 14 (C) by T_H-cells are shown. Expression of CD44/CD62L, IL-4 and IFN-γ in unimmunized control mice (D).

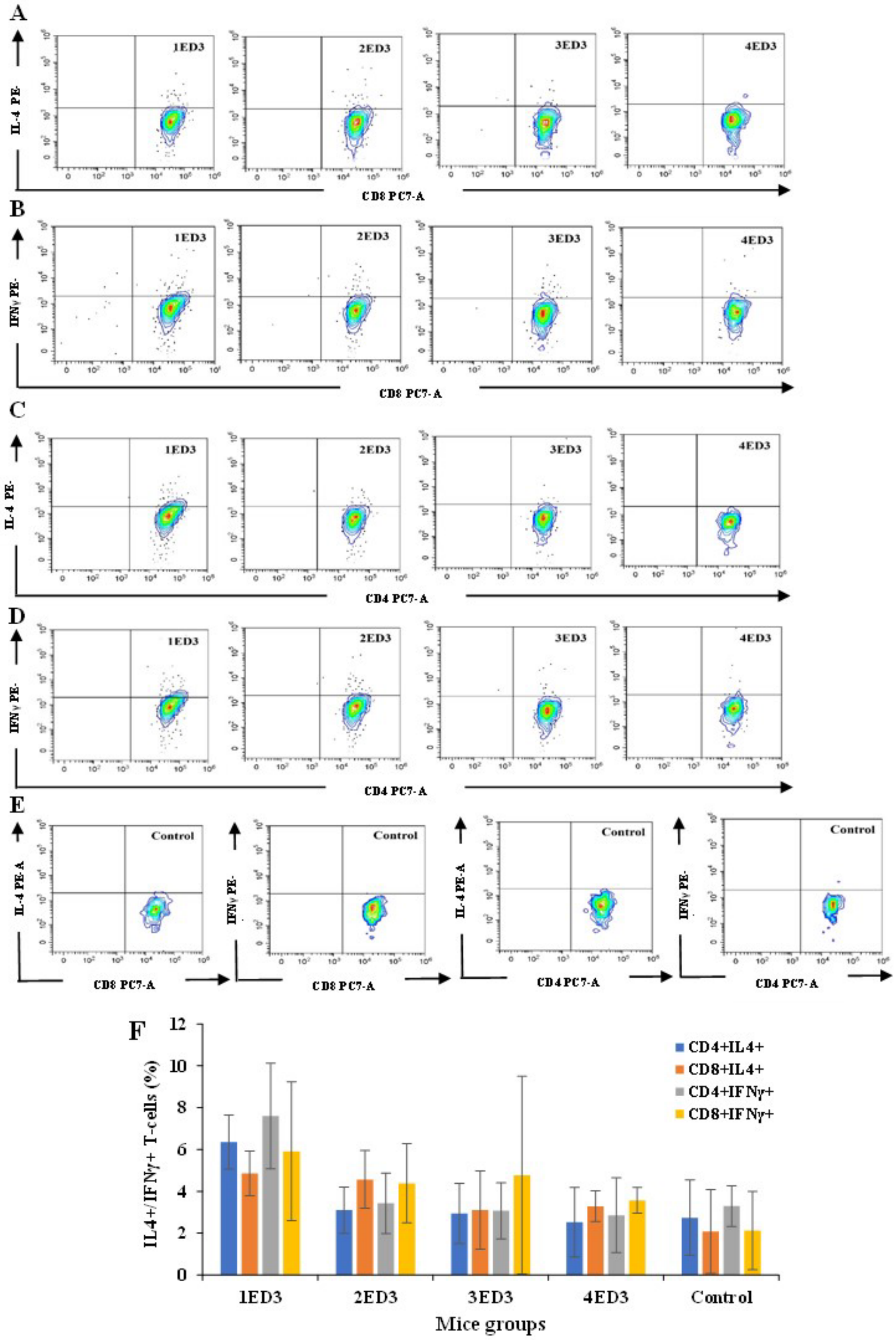

Figure S4. Effects of ED3 immunization on T-cell memory status in Balb/c mice. IL-4 expression (A) and IFN-γ expression (B) by T_H-cells and IL-4 expression (C) and IFN-γ expression (D) by T_H-cells at day 21 are shown. Expression of IL-4 and IFN-γ in unimmunized control mice (E). The relative percentages of CD44+CD62L+T-cells, and percentages of T-cells expressing IL-4 and IFN-γ in ED3 immunized mice are shown in panel (F).

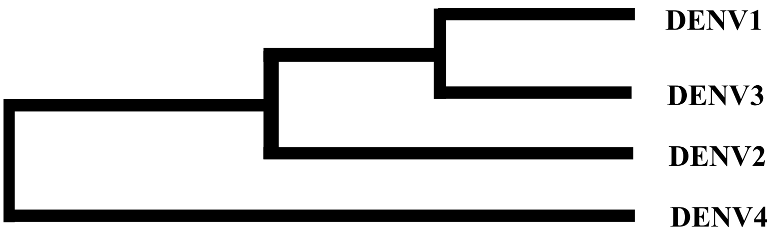

Figure S5. Phylogenetic tree topology of DENVs based on the ED3 sequences analyzed by maximum likelihood using CLUSTAL W [22]. Amino acid sequences of the four serotypes were used to reconstruct the phylogenetic tree and ancestral sequences.
